# Gene Ontology synonym generation rules lead to increased performance in biomedical concept recognition

**DOI:** 10.1186/s13326-016-0096-7

**Published:** 2016-09-09

**Authors:** Christopher S. Funk, K. Bretonnel Cohen, Lawrence E. Hunter, Karin M. Verspoor

**Affiliations:** 1 Computational Bioscience, University of Colorado School of Medicine, Aurora, CO 80045 USA; 2 Department of Computing and Information Systems, University of Melbourne, Parkville, Melbourne, 3010 Australia; 3 Health and Biomedical Informatics Centre, University of Melbourne, Parkville, Melbourne, 3010 Australia

**Keywords:** Biomedical concept recognition, Named entity recognition, Text-mining, Gene ontology

## Abstract

**Background:**

Gene Ontology (GO) terms represent the standard for annotation and representation of molecular functions, biological processes and cellular compartments, but a large gap exists between the way concepts are represented in the ontology and how they are expressed in natural language text. The construction of highly specific GO terms is formulaic, consisting of parts and pieces from more simple terms.

**Results:**

We present two different types of manually generated rules to help capture the variation of how GO terms can appear in natural language text. The first set of rules takes into account the compositional nature of GO and recursively decomposes the terms into their smallest constituent parts. The second set of rules generates derivational variations of these smaller terms and compositionally combines all generated variants to form the original term. By applying both types of rules, new synonyms are generated for two-thirds of all GO terms and an increase in F-measure performance for recognition of GO on the CRAFT corpus from 0.498 to 0.636 is observed. Additionally, we evaluated the combination of both types of rules over one million full text documents from Elsevier; manual validation and error analysis show we are able to recognize GO concepts with reasonable accuracy (88 %) based on random sampling of annotations.

**Conclusions:**

In this work we present a set of simple synonym generation rules that utilize the highly compositional and formulaic nature of the Gene Ontology concepts. We illustrate how the generated synonyms aid in improving recognition of GO concepts on two different biomedical corpora. We discuss other applications of our rules for GO ontology quality assurance, explore the issue of overgeneration, and provide examples of how similar methodologies could be applied to other biomedical terminologies. Additionally, we provide all generated synonyms for use by the text-mining community.

**Electronic supplementary material:**

The online version of this article (doi:10.1186/s13326-016-0096-7) contains supplementary material, which is available to authorized users.

## Background

The Gene Ontology (GO) represents the standard by which we refer to functions and processes that genes/gene products participate in. Due to its importance in biology and the exponential growth in the biomedical literature over the past years, there has been much effort in utilizing GO for text mining tasks [1, 2]. Performance on these recognition tasks is lacking; it has been previously seen that there is a large gap between the way concepts are represented in the ontology and the many different ways they are expressed in natural text [3–5].

There are two main applications of biomedical literature mining where improved recognition of Gene Ontology can improve downstream performance. 1) It is well known that manual curation can no longer keep up with the annotation of gene and protein function [6]. Automatic annotation is not our direct goal, but utilizing automatic methods to highlight functions could provide input to curators to help speed up manual curation. The more accurate automated methods become, the more useful their application becomes in manual curation. 2) The mining of GO concepts from large collections of biomedical literature has been shown to be useful for biomedical discovery, for example, pharmacogenomic gene prediction [7] and protein function prediction [8, 9]. Providing these discovery algorithms with not only cleaner, but more data, could increase the ability their accuracy of prediction and generalizability.

### Identification of gene ontology concepts in unstructured text

There are two main methods of identifying GO concepts within unstructured text, dictionary lookup and pattern/similarity based measures. Unfortunately, there have been very few evaluations assessing the ability to recognize and normalize Gene Ontology concepts from the literature; this is mostly due to lack of gold-standard annotations.

There are sub-tasks within the BioCreative I and IV [2, 10] community challenges that involve similar, but more involved, tasks to GO term recognition – relating relevant GO concepts given protein-document pairs. While the methods utilized for this specific tasks are beyond the scope of this work, some systems utilize these corpora to evaluate their ability to identify GO concepts on unstructured text. Ruch et al. [11] implement pattern based matching on a 5 token window and a vector space indexing model. Their GO pattern based matching reports highest average precision of 0.07 while their indexing model reports highest precision at recall = 0 (0.15) on the BioCreative I corpus. Gaudan et al. [12] utilize proximity, specificity, and similarity to calculate the score of GO term t appearing in zone z. They report average precision and recall of 0.34 for the terms at rank 1 on the BioCreative I corpus. A more recent system, GOCat [13], combines semantic similarity and a machine learning based *k*-NN algorithm to return the most similar *k* GO concepts in some text. On the Biocreative I corpus, GOCat reports 0.56 precision at recall 0.20 (F-measure = 0.29). A pitfall of these types of algorithms is they do not identify the exact span of text that matched the GO concept. They only specify that the concept could be present within this sentence or document.

Dictionary based methods identify the exact span of text that corresponds to the GO concept. Previous work evaluated concept recognition systems utilizing the Colorado Richly Annotated Full Text Corpus (CRAFT). Funk et al. [14] evaluated three prominent dictionary-based systems (MetaMap, NCBO Annotator, and ConceptMapper) and found Cellular Component was able to be recognized the best (F-measure 0.77). The more complex terms from Biological Process (F-measure 0.42) and Molecular Function (F-measure 0.14) were much more difficult to recognize in text. Campos et al. present a framework called *Neji* and compare it against *Whatizit* on the CRAFT corpus [15]; they find similar best performance (Cellular Component 0.70, Biological Process/Molecular Function 0.35). Other work explored the impact of case sensitivity and information gain on concepts recognition and report performance in the same range as what has previously been published (Cellular Component 0.78, Biological Process/Molecular Function 0.40) [16]. Since all previously published methods utilized dictionary based systems and report similar performance, there is a need for more sophisticated methods of utilizing the information contained within the Gene Ontology. For further progress to be made, the gap between concept representation and their expression in literature needs to be reduced, which serves as major motivation for the work presented in this manuscript.

There have been efforts to increase the ability to recognize biomedical concepts through enumerating variability in terms through generation, rewriting, and suppression rules. Tsuruoka et al. [17] generate spelling and punctuation variants based upon probabilistic generation rules learned from 84,000 MEDLINE abstracts. These types of rules help to capture the surface variability within concepts, such as “type I”, “Type I”, “type i”, etc. Hettne et al. [18] implement rewriting and suppression rules for to reduce the variability in UMLS concepts. For identification of terms, they remove leading parentheses/brackets and filter out some semantic types. Additionally, the suppress certain terms that should not be matched on, i.e. only EC numbers or those that contain dosages. While the rules presented here do not specifically utilize the methods described above, the same underlying principles are incorporated.

### Compositionality of the gene ontology

**Fig. 1:**
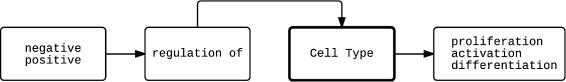
Finite state automata representing activation, proliferation, and differentiation GO terms. An abstracted FSA adapted from a figure in Ogren et al. [20] that shows how a particular term can be decomposed into its smaller components; where “cell type” can be any specific type of cell

The structure of concepts from the Gene Ontology has been noted by many to be compositional [19–21]. A term such as “GO:1900122 - positive regulation of receptor binding” contains another concept “GO:0005102 - receptor binding”; not only do the strings overlap, but the terms are also connected by relationships within the ontology. Ogren et al. explore more in detail terms as proper substring of other terms [19]. Additionally, previous work examined the compositionality of the GO and employed finite state automata (FSA) to represent sets of GO terms [20]. An abstracted FSA described in that work can be seen in Fig. 1. This example shows how terms can be decomposed into smaller parts and how many different terms share similar compositional structure. While using regular expressions are useful for simple terms, there are more complex concepts that require more sophisticated decomposition.

To facilitate generation of meaning (cross-product definitions) and consistency within the ontology, a system called *Obol* [22] was developed. This work involved analyzing the structure of terms through the creation of grammars to decompose and understand the formal language underlying the GO. An example grammar describing the positive regulation of a molecular function term follows: *process(P that positively_regulates(F)) ⇒ [positive],regulation(P),[of],molecular_function(F)*. These grammars serve as templates for the decompositional rules utilized in this work. Recently, GO has been moving away from pre-computed term, towards post-computed *on-the-fly* creation of terms for annotations using cross-products [23]. Additionally, TermGenie [24] was developed, using a pattern-based approach, to automatically generate new terms and place them appropriately within the Gene Ontology. This work dealt with the analysis and generation of new terms for curation, but no work has been focused on synonym generation.

There has been previous work using the compositional nature and common syntactic patterns within the Gene Ontology itself to automatically generate lexical elementary synonym sets [25]. This method generates a total of 921 sets of synonyms with a majority being generated from 1–3 terms; 80 % of the sets refer to orthographic {synthase, sythetase}, chemical products {gallate, gallic acid}, or Latin inflection {flagella, flagellum}. We believe this method is complementary to what we present here. In this work, we manually created these sets, along with many more, through analysis of Gene Ontology annotations in unstructured text. Additionally we go beyond and incorporate derivational variants, i.e. flagella ⇒flagellar, which have been shown to be very useful for capturing the natural language text of concepts. We were currently unable to find them publicly available, but if we should, the synonym sets by Hamon et al. could be seamlessly integrated within the synonym generation rules we present here.

Other work takes advantage of the structure of the Gene Ontology and relationships between GO terms to show that these properties can aid in the creation of lexical semantic relationships for use in natural language processing applications [26]. Besides compositionality, previous work tries to identify GO terms that express similar semantics that use distinct linguistic conventions [27]. They find, in general, that concepts from the Gene Ontology are very consistent in their representation (there are some exceptions but these are quality issues that the consortium would like to avoid or fix). The consistency of term representation along with the underlying compositional structure suggests the effective generation of synonyms for many terms using only a small number of rules.

### Current synonyms are not sufficient for text-mining

The identification of Gene Ontology terms is more difficult than many other types of named entities such as genes, proteins, or species mainly due to the length [14] and complexity of the concepts. To help illustrate this, we examined all variations of the GO term “GO:0006900 - membrane budding” within the CRAFT corpus. The entry for this concept in the ontology file is seen below. Like most other terms, the concept name appears as a noun and the entry contains a few synonyms (Table 1).

**Table 1:** Example ontology entry for the concept “membrane budding”

id: GO:0006900
name: membrane budding
namespace: biological_process
def: ~The evagination of a membrane resulting in formation of a vesicle.~
synonym: ~membrane evagination~ EXACT
synonym: ~nonselective vesicle assembly~ RELATED
synonym: ~vesicle biosynthesis~ EXACT
synonym: ~vesicle formation~ EXACT
is_a: GO:0016044 ! membrane organization and biosynthesis
relationship: part_of GO:0016192 ! vesicle-mediated transport

There were eight varying expressions of “membrane budding” in all of CRAFT, five of which are contained within a single article about expression and localization of Annexin A7 (PMID:12925238). In Table 2 we list the annotations along with sentential context. We find that using exact matching and context from the ontology file, the first two examples can be identified, but the others cannot. This one example illustrates that a rather simple term can be expressed in natural language text in many different ways, that convey identical semantic meaning.

**Table 2:** Examples of the “membrane budding” concept within a single document

Lipid rafts play a key role in **membrane budding**…
Having excluded a direct role in **vesicle formation**…
…involvement of annexin A7 in **budding of vesicles**
…Ca2+-mediated **vesiculation process** was not impaired
Red blood cells which lack the ability to **vesiculate** cause…

### Objectives of this work

We hypothesize that due to the highly formalized and compositional nature of the Gene Ontology [19], a small number of generation rules can help to automatically generate synonyms for current and novel GO concepts, differentiated from GO synonyms as “generated synonyms”. Additionally, we hypothesize that the variation captured in these generated synonyms will allow for better recognition of Gene Ontology concepts from the biomedical literature. We are aware that our method might overgenerate like Blaschke et al. [28], but we also hypothesize that those generated synonyms probably will not be found in the biomedical literature, and therefore, will not hinder performance.

In this work, we present 18 manually created rules to facilitate generation of synonyms from the entirety of the Gene Ontology. We evaluate these automatically generated synonyms both intrinsically, on a gold standard corpus, and extrinsically, through manual validation of annotations from a large literature collection. We show that these automatically generated synonyms increase recognition of GO concepts over any published results and illustrate the accuracy and impact the generated synonyms have at a large scale. Additionally, we show that the principles behind the rules generalizes to novel GO concepts. It is the goal to generate and release these generated synonyms for the larger biomedical natural language processing community. Currently, we do not suggest that all generated synonyms be considered for addition to GO, but filtering and classification methods could be employed to suggest the most accurate generated terms as synonyms. Not only does this work apply to the two tasks mentioned above, but it also adds the ability to generate synonyms for newly created GO concepts.

## Methods

### Methodological overview

The main idea behind our method is made up of three different steps:

1. Recursively decompose each Gene Ontology term to its constituent terms
2. Generate derivational variants for each of these constituent terms
3. Recombine all forms of all constituent terms (the constituent term itself, the generated derivational variants, and current synonyms of constituent term in the Gene Ontology) using differing syntactic and lexical rules

This methodology is made possible due to the highly formalized and compositional nature of the Gene Ontology.

Returning to the “membrane budding” example presented above (Table 2), we illustrate the methodology behind creation and application of our rules. By analyzing the different ways “membrane budding” is expressed in CRAFT, we find that a majority of the annotations are phrased around the end product, the vesicle. To help recognize these (currently) un-recognizable annotations there are two steps that should be done: 1) reorder words and change the syntax (“budding of vesicles”) and 2) generate derivational variants of “vesicle” (“vesiculation” and “vesiculate”). We developed two classes of rules that interact seamlessly to generate these types of synonym variation. The first we designate “recursive syntactic” and the second “derivational variant”, which are discussed immediately below. Each of our rules was manually created through the analysis of the differences between concept annotations within the gold standard CRAFT corpus and the Gene Ontology itself, along with discussions with an ontologist and biologist about how they most frequently express certain concepts. A more in-depth example is presented within the description of the individual rules.

#### Recursive syntactic rules

The recursive syntactic rules perform step 1 & 3 outlined in the “Methodological overview” section. The recursive rules, step 1, were developed through studying the grammars used in *Obol* [22], utilizing the dependency parse of the Gene Ontology terms from ClearNLP [29], and examining common formalizations within Gene Ontology concepts. These represent semi-frozen expressions as anchors to identify the constituent terms. The lexical and syntactic recombination rules, step 3, were derived by studying the transformations required to get from Gene Ontology term to the gold standard annotations that appear in CRAFT. Additionally, there were many discussions with biologists on the variation in terminology in which they could express the same concept.

We identified 11 cases when terms can be broken down into smaller composite terms; we acknowledge that there are more, but choose to focus on the ones that affected the majority of concepts. Over 55 % (14,221 out of 25,471) of the Gene Ontology concepts can be decomposed using at least one of these 11 different cases. Through our analysis we have developed an ordering for rule application, to generate the most possible synonyms. The 11 cases, the order, and examples applied are presented in Table 3; for full enumeration and further explanation of all rules see Additional file 1.

**Table 3:** Recursive syntactic rules order, constituent terms, and example generated synonyms

Order	Rule	GO term	Constituent terms	Generated synonyms
1	“via” or “involved in” terms	GO:0002679 - respiratory burst involved in defense response	“respiratory burst”, “defense response”	“defense response associated respiratory burst”
2	“regulation of” terms	GO:0030513 - positive regulation of BMP signaling pathway	“BMP signaling pathway”	‘positive regulation of BMP receptor pathway”, “up-regulation of BMP receptor signaling”
3	“response to” terms	GO:0034263 - autophagy in response to ER overload	“autophagy”, “ER overload”	“ER overload responsible for autophagy”, “autophagy response to ER overload”
4	“signaling” terms	GO:0035329 - hippo signaling	“hippo”	“hippo signaling pathway”, “signaling of hippo”
5	“biosynthetic process” terms	GO:0042095 - interferon-gamma biosynthetic process	“interferon-gama”	“interferon-gamma biosynthesis”, “production of interferon-gamma”
6	“metabolic process” terms	GO:0042120 - alginic acid metabolic process	“alginic acid”	“metabolism of alginic acid”, “alginic acid metabolism”
7	“catabolic process” terms	GO:0042190 - vanillin catabolic process	“vanillin”	“vanillin degradation”, “breakdown of vanillin”
8	“binding” terms	GO:0042314 - bacteriochlorophyll binding	“bacteriochlorophyll”	“binding of bacteriochlorophyll”, “bacteriochlorophyll bound”
9	“transport” terms	GO:0042876 - aldarate transmembrane transporter activity	“aldarate”, “transmembrane”	“transportation of aldarate across the membrane”, “transporting aldarate transmembrane”
10	“differentiation” terms	GO:0043158 - heterocyst differentiation	“heterocyst”	“heterocyst cell differentiation”, “differentiation into heterocyst”
11	“activity” terms	GO:0043492 - ATPase activity, coupled to movement of substances	“ATPase”, “coupled to movement of substances”	“ATPase, coupled to movement of substances”, “coupled to movement of substances activity of ATPase”

While these examples show only one rule applied at once, each constituent term identified recursively goes through each rule in the order outlined to determine the most basic constituent terms, which will get derivational variations (discussed in next paragraph) and then combinatorially re-combined into generated synonyms of the original term

#### Derivational variant rules

Once the original term is broken down to its constituent components, step 1, through the recursive syntactic rules presented above, we can apply derivational variant generation rules, step 2. The goal of this step is to generate synonyms that reflect the broader range of variability that occurs in natural language text expression of Gene Ontology concepts. We incorporate two open source tools to generate the derivational variants, WordNet [30] and Lexical Variant Generator [31]. There are a total of seven different specific cases when we apply these derivational generation rules (Table 4). These rules were developed by examining the transformations needed to create the text spans annotated in the CRAFT gold standard from the information contained within the GO. For example, for *single word terms* we would generate both verb and adjective forms of the noun concept, if they exist, which would then both be incorporated compositionally within the more complex concepts. For additional explanation and full enumeration of the rules see Additional file 1.

**Table 4:** Individual derivational variant generation rules

Order	Rule	Rule defined	GO terms	Example derivations
1	Single word terms	1 {NN} ⇒ {JJ}	1 GO:0043066 - negative regulation of **apoptosis**	1 “**apoptotic** down regulation”
		2 {NN} ⇒ {VB}	2 GO:0023040 - **signaling** via ionic flux	2 “**signaled** via ionic flux”
2	Double word terms	1 {NN_1 NN_2} ⇒ {NN_1}, {VB_2 NN_1}, {JJ_1 NN_2}, {NN_1 JJ_2}	1 GO:0048666 - **neuron** development	1 “**neural** development”, “**neurotic** development”, “**neuronal** development”
		2 {JJ_1 NN_2} ⇒ {JJ_1}, {JJ_1 JJ_2}	2 GO:0005576 - **chromosomal** region	“**chromosomal**”, “**chromosome** region”
3	Triple word terms	1 {NN_1 NN_2 NN_3} ⇒ {NN_1 NN_3}, {NN_3 NN_1}, {VB_3}	1 GO:0052386 - cell wall **thickening**	1 “**thickened** wall”, “cell**thickening**”, “**thickens** cell wall”
4	“cell part” terms	Introduce and re-order cell part terms	GO:0035452 - **extrinsic component** of plastid membrane	“**peripheral** to plastid membrane”, “**extrinsic** to plastid membrane”
5	“sensory perception” terms	Introduce variants of the sense - “sensory perception of {NN}”	GO:0050909 - sensory perception of **taste**	“**gustory**”, “**gustation**”
6	“transcription, *X*-dependent” terms	Introduce variants of “transcription”	GO:0006410 - **transcription**, RNA-templated	“RNA-dependent **reverse transcription**”, “RNA-dependent **RT**”
7	“*X* strand annealing activity” terms	Introduce variants of “annealing”	GO:0033592 - RNA strand **annealing** activity	“RNA **hybridization**”, “**hybridize**”

The seven patterns that we generate derivational variants are presented along with examples of each. While these are presented individually, all derivational and recursive syntactic (presented in Table 3) interact at each step. The examples provided are single GO terms, but any of the constituent terms produced through the above steps will go through all derivational rules, if possible. The bolded words in the GO Term and Synonyms generated column represent the impact of the rule. The Penn Treebank part-of-speech (POS) tags are utilized below: *NN* = noun, *VB* = verb, *JJ* = adjective. All varying forms were converted to the basic POS tag, e.g. *NNS* = plural noun and were converted to NN

#### Example of rules applied

**Fig. 2:**
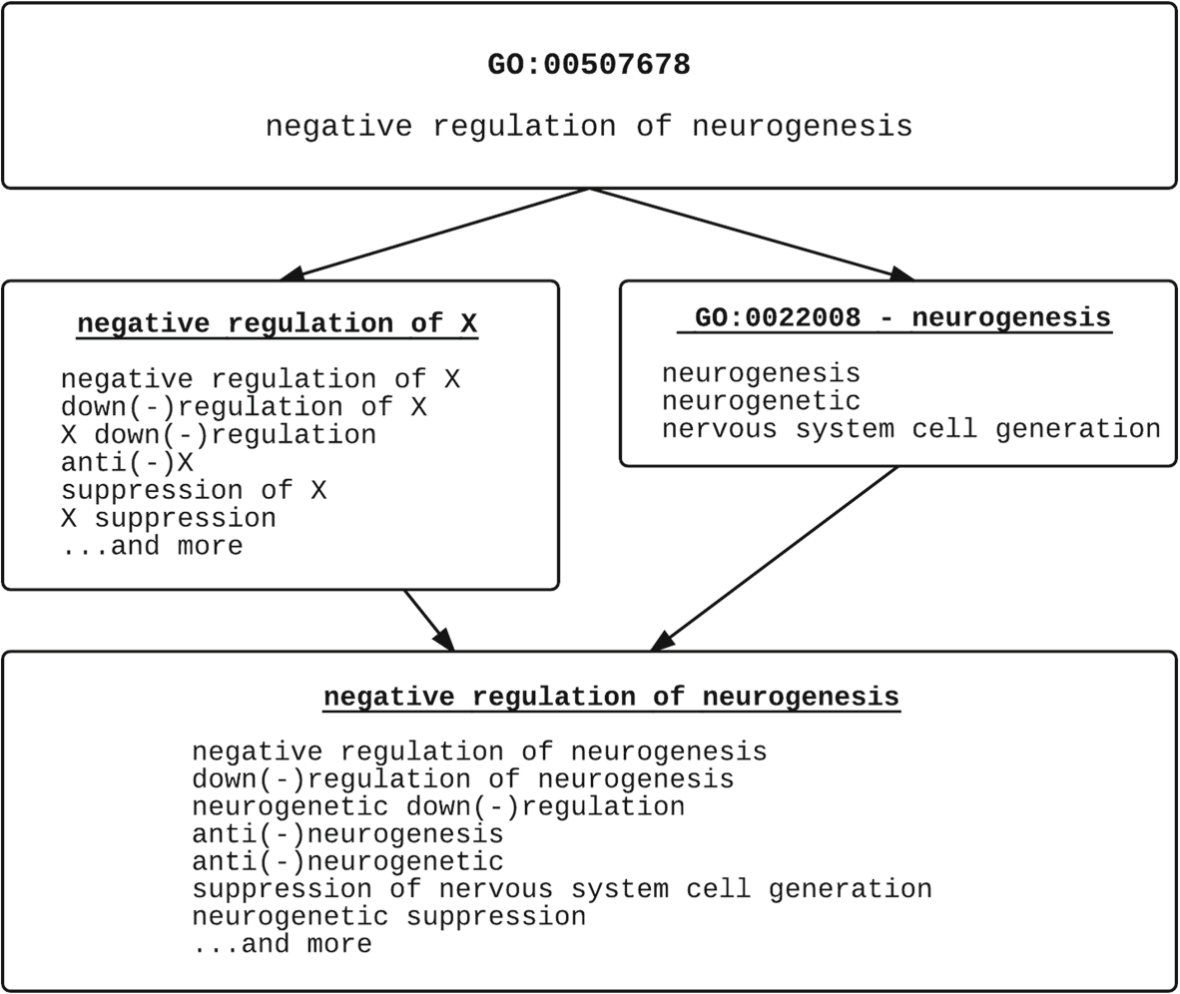
Three steps of synonym generation applied. A single GO concept broken down into its composite parts (*bolded* and *underlined*), synonyms generated for each part (text underneath the part), then combination of all synonyms from all composite parts to form complete synonym of the original concept

In Fig. 2 we walk through all three steps of the synonym generation process with the concept “GO:00507678 - negative regulation of neurogenesis”.

1. It is decomposed into 2 constituent terms: 1) “negative regulation of” and 2) another GO concept – “GO:0022008 - neurogenesis”. Since it cannot be decomposed any further, we begin generating synonyms for both of these composite parts.
2. Derivational variants for the term “neurogenesis” are generated utilizing the *single word term* rule. There are three different forms of “neurogenesis”, the term itself, the adjective form exists in WordNet [30] or can be generated through LVG (lexical variant generator) [31], and the current synonyms found within the Gene Ontology.
3. There are no derivational variants of “negative regulation of”, but there are syntactic and lexical synonymous expressions enumerated in the *“regulation of” terms* rule. To generate synonyms of the original concept, the three forms of “neurogenesis” are combinatorially combined with the 12 different synonymous expressions of “negative regulation of” to form 36 synonyms for the original term; the Gene Ontology currently only has 4 synonyms for this concept.

### ConceptMapper

ConceptMapper (CM) is an open source highly configurable dictionary lookup tool created for identifying named entities in text. CM is part of the Apache UIMA Sandbox [32] and is available at http://uima.apache.org/d/uima-addons-current/ConceptMapper. Version 2.3.1 was used for these experiments.

The first step in the CM pipeline is to convert the GO ontologies to the required XML dictionary format. The document text is then provided and tokenized. All tokens within a span, in this case a sentence, are looked up in the dictionary using a configurable lookup algorithm. The lookup algorithm has the ability to reorder words, insert gaps, ignore words, identify all or only longest match, etc. For each branch of GO we used the highest performing parameter combination previously identified [14]. Additional file 2 provides a summation of the different type of ConceptMapper parameters and shows the exact parameter combinations used for recognition of each sub-branch of the Gene Ontology.

### Concept recognition pipeline and baselines

The baseline for GO recognition was established in previous work [14] through parameter analysis of three different concept recognition systems. The top performing system, ConceptMapper (CM), is used for the following test because it produced the highest F-measures on 7 out of 8 ontologies in the CRAFT corpus. CM takes an obo file and converts it to an xml dictionary, which is used to recognize concepts in free text. In analyzing the results there are two different baselines that were provided. Both baselines use the same ConceptMapper parameters settings but differ in the way the dictionary was created:

- B1, a CM dictionary containing only information within the ontology obo file.
- B2, a CM dictionary that deletes the word “activity” from molecular function terms containing that word (for example, for term “GO:0016787 - hydrolase activity” a synonym of “hydrolase” is added). This addresses a known property of molecular function terms formalization that aims to separate of the protein and the function of the protein.

For the intrinsic evaluation pipeline on the CRAFT corpus, we use the version of GO used to annotate CRAFT from November 2007. We are aware of the great number of changes made, but this was purposefully done to keep the concepts available to the dictionary the same that were available to the annotators when they marked up the gold standard. To show that the rules created are able to generalize and apply to the many new concepts added to the Gene Ontology added since 2007, for the extrinsic evaluation on large collection we use an updated version of the GO from 9/25/2015.

### Evaluation corpora

There are two different corpora utilized in evaluation of our generated synonyms.

#### CRAFT corpus

The gold standard used is the Colorado Richly Annotated Full-Text (CRAFT) Corpus [33, 34] version 1.0 released October 19th, 2012. The full CRAFT corpus consists of 97 completely manually annotated biomedical journal articles, while the “public release” set, which consists of 67 documents, was used for this evaluation. CRAFT includes over 100,000 concept annotations from eight different biomedical ontologies. Even though the collection is small relative to the size of PubMed, there is no other corpus that has text-level annotations of Gene Ontology concepts.

#### Large literature collection

To test generalization and for further analysis of the impact our concept recognition can have, we utilized a large collection of one million full-text articles from Elsevier. This is a collection of full-text documents from a wide variety of biomedical Elsevier journals that was delivered to the University of Colorado for internal analysis.

### Evaluation of generated synonyms

To evaluate the synonyms given we use the same pipelines described in Funk et al. [14]. Synonyms are generated by each method and then only those that are unique (both within the generated synonyms and GO itself) are inserted into a temporary obo file. The temporary obo file is then used to create an xml dictionary used by ConceptMapper [35] for concept recognition. The CRAFT corpus is used as the gold standard and precision, recall, and macro-averaged F-measure are reported for each branch of the GO. We provide counts of concepts along with changes from the evaluation on the large scale corpus.

## Results and discussion

### Application of gene ontology synonym rules

To explore the impact that our rules had on the recognition of concepts from the biomedical literature, we applied our synonym generation rules to two different version of the Gene Ontology and compared the concepts identified before/after application on two different biomedical corpora. For evaluation on the CRAFT corpus, we applied our rules to the CRAFT annotated version of GO containing 25,471 concepts; our 18 rules generated 291,031 synonyms for 16,800 concepts (66 % of all concepts). Because the CRAFT version is from 2007, we applied our methodology to a more recent version of GO from September 2015. On this recent version, our rules generated ∼1.5 million unique, but unconfirmed, synonyms for 66 % of all GO concepts (27,610 out of 41,852). Only a few rules, 18, can have wide applicability to a majority of the concepts in the Gene Ontology due to the concepts being highly formalized and exhibiting a compositional nature. While our rules appear to overgenerate, the main focus of this work is to improve recognition of GO concepts from the biomedical literature; we expect overgeneration to not decrease performance because a majority of the generated synonyms will not be seen in the biomedical literature. An easy method of reducing the overgeneration would be to only include the generated synonyms that currently appear in all of MEDLINE or through an exact Google search.

Not only do the introduced rules generate new synonyms, but are also able to recreate 67 % of all synonyms (68,174 out of 101,615) from all concepts on the 2015 version. This illustrates the usefulness of our presented methodology for not only synonym generation for ontology curation and enhancement. We now focus on how introducing variation through synonym generation aids in identification of Gene Ontology concepts from the biomedical literature.

### Synonym evaluation on a gold standard

The overall results for all methods performance on CRAFT can be seen in Table 5 with more detailed analysis of each method following. More details about how we evaluated performance of each method can be seen in *Evaluation of generated synonyms*.

**Table 5:** Micro-averaged results for each synonym generation method on the CRAFT corpus

Method	TP	FP	FN	Precision	Recall	F-measure
Baseline (B1)	10,778	6,280	18,669	0.632	0.366	0.464
Baseline (B2)	12,217	7,367	17,230	0.624	0.415	0.498
All external synonyms	12,747	11,682	16,704	0.522	0.433	0.473
Recursive syntactic rules	12,411	7,587	17,036	0.621	0.422	0.502
Recursive syntactic and derivational rules	18,611	10,507	10,836	0.639	0.632	**0.636**

Bold highlighting indicates the method that produces the highest F-measure

Besides the rules presented, there are a number of manually curated external mappings from Gene Ontology concepts to other data sources such as UniProt [36], the Brenda database [37], and Wikipedia [38]. To test the usefulness of these mappings as sources of synonyms, we imputed synonyms for the Gene Ontology concept from synonyms of the linked concept in the respective data source. Overall, we find that external ontological mappings introduce significantly more errors than correctly recognized concepts and are not suggested to be useful, in their current form, as a whole, for concept recognition (methods and detailed analysis of each data source can be seen in Additional file 3).

Overall, the best results are obtained by using both syntactic recursive and derivational rules; an increase in F-measure of 0.112 is seen (0.610 vs 0.498). This performance gain is the result of a large increase in recall (0.225) with only a modest decrease in precision (0.049). Examining the overall performance we find that all methods perform better than B1, while all but the external synonyms perform better than B2. Overall, all generation methods increase recall with a decrease in precision, which is to be expected when adding synonyms. We now discuss the impact of synonyms generated through both classes of rules.

#### Performance impact of generated synonyms

The Gene Ontology is broken down into three sub-ontologies, Cellular Component (CC), Biological Process (BP), and Molecular Function (MF). Terms from each sub-ontology have differing biological meaning and textual characteristics – some rules are more applicable to one sub-ontology than another, so we evaluate them separately. We apply only the recursive syntactic rules (Steps 1 & 3, described in “Methodological overview”) to all concepts within the Gene Ontology and evaluate on the full-text CRAFT corpus using our dictionary based lookup system ConceptMapper; performance can be seen in Table 6. For Cellular Component, only a few new synonyms are generated, which is not surprising, because concepts from this branch normally do not appear compositional in nature. These new CC have no impact when compared to the baselines.

**Table 6:** Performance of manual Gene Ontology rules on the CRAFT corpus

Method	Generated synonyms	Affected terms	TP	FP	FN	P	R	F
Cellular Component (CC)								
Baseline (B1)	X	X	5,532	452	2822	0.925	0.662	0.772
Baseline (B2)	X	X	5,532	452	2822	0.925	0.662	0.772
Syntactic recursion rules	23	21	5,532	452	2,822	0.925	0.662	**0.772**
Both rules	4,083	724	6,585	969	1,769	0.872	0.788	**0.828**
Molecular Function (MF)								
Baseline (B1)	X	X	337	146	3,843	0.698	0.081	0.145
Baseline (B2)	X	X	1,772	964	2,408	0.648	0.424	0.512
Syntactic recursion rules	11,637	7,353	1,759	977	2,421	0.643	0.421	0.509
Both rules	14,413	7,401	2,422	1,074	1,758	0.693	0.579	**0.631**
Biological Process (BP)								
Baseline (B1)	X	X	4,909	5,682	12,004	0.464	0.290	0.357
Baseline (B2)	X	X	4,913	5,951	12,000	0.452	0.291	0.354
Syntactic recursion rules	182,617	6,847	5,120	6,158	11,793	0.454	0.303	**0.363**
Both rules	272,535	8,675	9,604	8,464	7,309	0.532	0.568	**0.549**
All Gene Ontology								
Baseline (B1)	X	X	10,778	6,280	18,669	0.632	0.366	0.464
Baseline (B2)	X	X	12,217	7,367	17,230	0.624	0.415	0.498
Syntactic recursion rules	194,277	14,221	12,411	7,588	17,036	0.621	0.422	**0.502**
Both rules	291,031	16,800	18,611	10,507	10,836	0.640	0.632	**0.636**

Bold highlighting indicates where the generated synonyms have a positive effect on the performance

Eighty six percent (7,353 out of 8,543) of terms within Molecular Function had at least one new synonym generated by the recursive syntactic rules. Unexpectedly, performance on MF slightly decreases. The performance on Biological Process slightly increases with the addition of recursive syntactic rules. BP sees the largest increase in the number of new synonyms generated, with over 180,000 new synonyms for 46 % (6,847 out of 14,767) of BP concepts. The syntactic recursive rules are most helpful in generating Biological Process synonyms that match instances within CRAFT. For example, 74 more correct instances of “GO:0016055 - Wnt receptor signaling pathway”, expressed in the gold standard as “Wnt signaling” and “Wnt signaling pathway”, are able to be identified. These are generated through the *signaling terms* rule which found that both the words “receptor” and “pathway” were uninformative.

MF and BP share similarities in the kinds of errors introduced: a true positive (TP) in the baseline is converted to a false positive (FP) and false negative(s) (FN) because a longer term is identified through one of the generated synonyms (one ConceptMapper parameter used specifies that only the longest match is returned). It is possible that these are missing annotations within the gold standard. For example, one of the generated synonyms for “GO:0019838 - growth factor binding” is “binding growth factor”. In the corpus, “bound growth factor” is annotated with both “GO:0005488 - binding” and “GO:0008083 - growth factor activity”. With our generated synonyms added to the dictionary, the same text span is only annotated with the more specific “GO:0019838 - growth factor binding” which results in the removal of two true positives and the introduction of one false positive, thus reducing overall performance, but possibly increasing the accuracy of annotations. If this is a wide-spread issue, changing the parameters for our dictionary lookup will allow it to find all concepts, which would identify all three annotations instead of only the longest one.

Overall, despite the decrease in performance of Molecular Function terms, the recursive syntactic rules slightly improve concept recognition of the Gene Ontology on the CRAFT corpus over baseline 2 (∼200 more TPs and ∼200 more FPs introduced). Because the CRAFT corpus contains only a small portion of the whole GO (1,108) and these rules only account for reordering of tokens within GO, we did not expect to see a large increase in concept recognition performance.

When we apply both the recursive syntactic and derivational rules (Steps 1, 2 & 3, described in “Methodological overview”) to all concepts and evaluate on the full-text CRAFT corpus we see improvements for all branches (Table 6). (The derivational rules cannot be evaluated on their own due to an implementation dependency to the recursive syntactic rules. The derivational rules assume that all concepts passed in will already be decomposed into their smallest GO components. The real power comes when combining both rules because variation is being introduced in only parts of the longer GO concepts.) Each branch has different properties and when evaluated individually, we see an increase in F-measure for all. This increase is due to a large gain in recall (up to 0.27). For both Biological Process and Molecular Function, precision also increases, while precision slightly decreases for Cellular Component. When performance is aggregated over all branches of the Gene Ontology, an increase in F-measure of 0.14 (0.498 vs. 0.636) is seen; this comes from both an increase in recall (0.22) and precision (0.02). Our rules introduce ∼291,000 generated synonyms which cover 66 % (16,800 out of 25,471) of all terms within GO.

#### Analysis of generated synonyms

Now we explore which generated synonyms contribute the most to the increase in performance seen on the gold standard corpus. The top 5 concepts that impact these performance numbers are presented in Table 7. For Cellular Component, the most helpful synonym generated “immunoglobulin” ⇒“antibody” is seen many times within CRAFT and is contained within the *double word* rule. The other four are generated using the *single word* rule, specifically converting from the noun form from the ontology to the adjective form. Through examining Molecular Function terms, it became clear that “annealing” was missing synonymous representation within the Gene Ontology; within the *annealing* rule we add a synonym of “hybridization”. Two of the next most helpful synonyms are due to excluding low information containing words and derivational variations. It should be noted that within Molecular Function an even larger increase in performance is seen between baseline 1 and 2 (Table 6), which takes into account the many “activity” terms. These types of synonyms are also accounted for in our rules and are compositionally combined into other terms. For Biological Process we observe that the most helpful synonyms are generated using the *double word* and *single word* derivational rules. We also find that generating different lexical forms of both single word concepts and within longer terms helps to introduce many true positive annotations.

**Table 7:** The top 5 derivational synonyms that improve performance on the CRAFT corpus

GO ID	Term name	*Δ*TP	*Δ*FP	*Δ*FN	Generated synonyms
Cellular Component					
GO:0019814	Immunoglobulin complex	+548	+0	−548	Antibody, antibodies
GO:0005634	Nucleus	+218	+35	−218	Nuclear, nucleated
GO:0005739	Mitochondrion	+135	+0	−135	Mitochondrial
GO:0031982	Vesicle	+11	+3	−11	Vesicular
GO:0005856	Cytoskeleton	+15	+0	−15	Cytoskeletal
Molecular Function					
GO:0000739	DNA strand annealing activity	+327	+1	−327	Hybridized, hybridization, annealing, annealed
GO:0033592	RNA strand annealing activity	+327	+1	−327	Hybridized, hybridization, annealing, annealed
GO:0031386	Protein tag	+6	+79	−6	Tag
GO:0005179	Hormone activity	+1	+0	−1	Hormonal
GO:0043495	Protein anchor	+1	+10	−1	Anchor
Biological Process					
GO:0010467	Gene expression	+2235	+361	−2235	Expression, expressed, expressing
GO:0007608	Sensory perception of smell	+445	+1	−445	Olfactory
GO:0008283	Cell proliferation	+97	+71	−97	Cellular proliferation, proliferative
GO:0007126	Meiosis	+93	+2	−93	Meiotic, meiotically
GO:0006915	Apoptosis	+173	+2	−173	Apoptotic

The GO terms that increase performance the most on CRAFT are along with the change (*Δ*) in number of true positives (TP), false positives (FP), and false negatives (FN) from the baseline B2 (“activity” removed baseline). The generated synonyms that result in this increase are shown under ‘Generated synonyms’

From examining the top most helpful synonyms, we provide evidence that the derivational synonyms improve performance on a manually annotated corpus through the introduction of more linguistic variability, which decreases the gap between concepts in the ontology and their expression in natural language text. Overall, the top generated synonyms that improve performance do not take into account much of the compositional nature of GO terms. We believe this is due to two aspects; 1) The annotation guidelines used to define what constitutes a correct mention of a GO concept in CRAFT [39] and 2) CRAFT is only a small representation of what is contained within the entire biomedical literature. This small representation is due to the paper content (only mouse papers resulting in functional annotation of at least one protein), small corpus size, and appearance of only a small subsection of the Gene Ontology. To further evaluate the synonyms generated by our rules without the aforementioned drawbacks, in the next section, we explore the impact our rules make on a large collection of the biomedical literature.

### Evaluation of generated synonyms on a large full text collection

We evaluated the impact of synonyms generated by both recursive syntactic and derivational variant rules have on the ability to recognize GO concepts within a large collection of one million full text documents. Unlike the previous evaluation, these documents do not have any manual annotation or markup of Gene Ontology concepts, so we are unable to calculate precision/recall/F-measure. However, we can calculate descriptive statistics and perform manual evaluation of a random sample of the differences in annotations produced when our rules are applied. For these we used a version of GO from September 2015. Applying our rules generates ∼1.5 million new synonyms for 66 % of all GO concepts (27,610 out of 41,852).

Since one of the primary focuses of the Gene Ontology is functional annotation of proteins, we imparted some of that knowledge into the large scale analysis by calculating information content of each concept with respect to the experimental UniProt GOA annotations [40]. We calculated the information content (IC) described in Resnik et al. [41]. The IC scores range from 0-12.25; a lower score corresponds to a term that many proteins are annotated with and should appear many times in the literature while a high scoring term is more specific and might have only one or two annotations in GOA. For example, a common term such as “GO:0005488 - binding” has a score of 0.80 while a more informative term “GO:0086047 - membrane depolarization during Purkinje myocyte cell action potential” has a score of 12.25. A score of “undefined” corresponds to a concept that is not currently annotated to any protein with GOA. It is our hypothesis that the most informative terms (higher IC) would be more difficult to identify in text and that our rules, described above, would help increase the frequency at which we can recognize correct mentions of these highly informative terms.

Statistics for both the concepts recognized using the ontology (baseline 2 presented above) and rules applied along with the differences broken down by information content can be seen in Table 8. Utilizing only the information contained within the Gene Ontology, and accounting for “activity” terms, ∼97 million mentions of ∼12,000 unique GO concepts are identified. After generation of synonyms by both the recursive syntactic and derivational rules, ∼138 million mentions of ∼14,100 unique GO concepts are identified. In summation, our rules aid in the recognition of ∼41 million more mentions for all GO concepts (∼42 % increase) along with the ability to recognize ∼2,000 unique GO concepts (∼18 % increase) that are not previously identified using the ontology alone. There were a total of ∼2.5 million mentions associated with the 2,135 unique concepts that were only found when the synonym generation rules were applied. The other ∼39 million new mentions are associated with the ∼12,000 concepts both dictionaries recognize.

**Table 8:** Statistics of annotations produced on the large literature collection by information content

	Baseline B2		With generated synonyms		Impact of synonyms		
IC	# Terms	# Annotations	# Terms	# Annotations	New concepts	New annotations	Change
Undefined	3,548	16,929,911	4,303	23,653,066	755	6,723,155	+39.7 %
[0,1)	7	3,202,114	7	3,177,333	0	−24,781	−0.1 %
[1,2)	16	2,655,365	17	2,801,431	1	146,066	+0.1 %
[2,3)	43	7,332,003	44	8,016,573	1	684,570	+0.1 %
[3,4)	94	4,474,422	101	5,188,968	7	714,546	+0.2 %
[4,5)	178	4,185,438	191	9,340,757	13	5,155,319	+123.8 %
[5,6)	354	13,547,423	373	22,284,670	19	8,737,247	+64.4 %
[6,7)	666	9,533,940	715	12,060,499	49	2,526,559	+26.3 %
[7,8)	1,044	18,354,299	1,154	21,251,834	110	2,897,535	+16.8 %
[8,9)	1,465	7,932,937	1,648	15,316,476	183	7,383,539	+92.4 %
[9,10)	1,551	4,813,153	1,813	7,671,601	262	2,858,448	+58.3 %
[10,11)	1,396	2,390,061	1,690	4,291,831	294	1,901,770	+79.1 %
[11,12)	942	1,246,758	1,162	2,279,005	220	1,032,247	+83.3 %
[12,13)	732	578,501	953	1,257,956	221	679,455	+117.2 %
Total	12,036	97,176,325	14,171	138,592,000	2,135	41,415,675	+42.5 %

Shows the number of unique terms and total number of annotations produced through baseline B2, both derivational and syntactic recursive rules applied, and the impact the rules have overall. The change is percent change in total annotations

The biggest increase in number of annotations and concepts identified can be seen in those concepts with undefined and higher information content (IC > 8). This shows that the our syntactic and derivational rules successfully introduce variation that allow the more specific and information containing concepts to be recognized either at all or more frequently. While we do not find much change in annotations produced on the lower information content concepts, we do see a negative change in annotations produced for some of the low information containing concepts. This is due to our rules generating synonyms that can help to identify more specific concepts. For example, “GO:0005215 - transporter activity” is found ∼75,000 fewer times after the addition of our generated synonyms due to more specific transporters being identified. For instance, in the following sentence, the bold text corresponds to the concept recognized using the baseline, while the italicized concept is exactly generated through the use of our rules: “The present study was aimed to evaluate whether intraperitoneal carnitine (CA), a ***transporter****of fatty acyl-CoA* into the mitochondria….” (PMID: 17239403). The usefulness of these rules goes beyond that of just improving our recognition of concepts from test as identification of more informational GO concepts has been shown to increase performance on the protein function prediction task [8, 9].

Examining the overall numbers of concepts and mentions recognized provides insights into how useful the synonyms generated are for recognition of GO concepts from the biomedical literature. Since most mentions identified using only the ontology information were also found when the rules were applied, this indicates that our rules aid in identification of many new concepts along with new mentions of concepts, thus leading to an overall increase in recall. We saw in evaluation on CRAFT that both precision and recall were increased; we explore through manual validation the accuracy of concepts identified utilizing the generated synonyms on a large scale in the following section.

#### Manual validation of gene ontology mentions

Although we found an improvement in performance on the CRAFT corpus and on the larger corpus a significant number of additional concepts and mentions were identified through our synonym generation rules, we are hesitant to reach any further conclusions without some manual validation of the accuracy of these generated synonyms. There are too many concepts and annotations produced to manually validate them all, so we performed validation of a randomly distributed subset of concepts and instances of those concepts within text. For cases where the validity of the term was unclear from the matched term text alone we went back to the original paper and viewed the annotation in sentential context. For a baseline of performance, we validated a random sample of 1 % of baseline concepts (125 concepts with ∼1,200 randomly sampled mentions) from each IC range and a random sample of 10 % of all new concepts (217 terms with ∼1,450 randomly sampled mentions) recognized through our rules; these results are presented in Table 9. We find that overall accuracy is very high (0.94) for the concepts recognized only utilizing the ontology information. A majority of these text spans identified are exact, or very near, matches to the official ontological name or one current synonyms. The only variation introduced is through a stemmer or lemmatizer used in the concept recognition pipeline (see Additional file 2 for more details). The annotations produced when using synonyms generated through our rules do not have as high of accuracy (0.74) but still produce reasonable results.

**Table 9:** Results of manual inspection of random samples of annotations

	Baseline B2			With rules			Overall
IC	# Terms	# Annotations	Accuracy	# Terms	# Annotations	Accuracy	Accuracy
Undefined	35	231	0.98	75	363	0.70	0.81
[0,1)	1	15	0.20	0	0	0.00	0.20
[1,2)	1	15	1.00	1	4	1.00	1.00
[2,3)	1	15	1.00	1	4	1.00	1.00
[3,4)	1	4	1.00	1	1	0.00	0.80
[4,5)	2	30	0.60	2	24	0.88	0.72
[5,6)	4	60	0.97	2	13	0.23	0.84
[6,7)	7	79	0.99	5	41	0.49	0.82
[7,8)	10	136	0.89	11	116	0.65	0.78
[8,9)	15	197	0.98	19	163	0.83	0.91
[9,10)	16	175	0.97	26	205	0.79	0.87
[10,11)	14	119	0.83	30	217	0.80	0.81
[11,12)	10	103	0.97	22	141	0.77	0.86
[12,13)	8	93	0.98	22	156	0.72	0.82
Total	125	1272	0.94	217	1448	0.74	0.83

Accuracy, calculated via manual review of textual annotations for correctness, of random subsets of concepts recognized from the large literature collections. We sampled 1 % of concepts, with up to 15 randomly sampled specific text spans per concept, from concepts identified using baseline B2. We sampled 10 % of concepts, with up to 15 randomly sampled text spans per concept, from the new concepts recognized through the presented synonym generation rules. Overall accuracy is calculated by combining annotations of the same IC from baseline and with our rules

Earlier, we hypothesized that overgeneration of synonyms would not hinder performance because synonyms that contain incorrect syntactic format or those that are not lexically sound, would not appear within the text we are searching. While performing manual evaluation of annotations produced, we noted that a majority of the errors came from three scenarios: 1) naive stemming introducing incorrect concepts (60 %), 2) incorrect level of specificity due to information loss (25 %), and 3) inclusion of incorrect punctuation (15 %). A detailed error analysis along with strategies to correct them is presented in Additional file 4. Based upon these results, we do not believe that the 1.5 million new synonyms generated introduce many false positives from overgeneration. While we see a decrease in accuracy in annotations returned from text when we include the synonyms generated by our rules, we do not attribute the decrease entirely to the synonyms themselves, as over half of the errors are due to interaction of synonyms and the stemmer utilized for dictionary lookup (Additional file 4). An interesting observation is that sometimes generating a phrase or synonym that initially appears incorrect can actually aid in recognition. An example is the different adjective forms of “protein”; most would use the form “proteinaceous”, but another form is generated through Lexical Variant Generator (LVG), “protenic”. This appears multiple times within articles translated into English, for example, the concept “GO:0042735 - protein body” is seen within the following sentence “The activity is exhibited through a **protenic body** of NBCF…” (PMID: 1982217).

### The impact of supercomputing on concept recognition tasks

We ran the our concept recognition pipeline over the large full text collection on the Pando supercomputer located at the University of Colorado, Boulder campus. It has 60 – 64 core systems with 512 GB each along with 4 – 48 core systems with 1TB ram each, for a total of 4,032 compute nodes. We utilized a quarter of the machine and ran our pipeline over 1,000 directories with 1,000 full text documents in each. We were able to produce GO annotations for all one million documents in around 10 minutes. Granted, no components are particularly complicated. They consist of a sentence splitter, tokenizer, stemmer/lemmatizer, followed by dictionary lookup, but we have performed similar tasks on a large memory machine, with 32 cores and the complete task has taken 3–4 weeks. Given that Pubmed consists of over 24 million publications, if it was possible to obtain all documents and performance is linear to the number of documents, we could recognize GO concepts from the entirety of the biomedical literature in around 4 hrs. More complex and time consuming tasks, such as relation extraction, will take longer but will still be on the order of days or weeks utilizing the power of a supercomputer, since these tasks are “embarrassingly parallel”.

### Generalization to other biomedical ontologies

The synonym generation methodology presented here, of breaking down complex concepts into their most constituent parts, generating synonym for the parts, then recursively combining to form synonyms of the original concept is one that can generalize to many other ontologies or standardized terminologies. The Gene Ontology contains very complex and lengthy worded concepts; the rules required to implement compositional synonyms in other ontologies might not need as many syntactic and derivational rules as we present here. Besides GO we can envision similar methodologies easily applied to Human Phenotype Ontology (HPO), Chemical Entities of Biological Interest (ChEBI), SNOMED, and International Classification of Diseases 10 (ICD10).

One example, within the Human Phenotype Ontology (HPO) [42], there is a high level HPO term that corresponds to “phenotypic abnormality”. There are just over 1,000 terms (∼10 % of all HPO concepts) that are descendants of “phenotypic abnormality” that can be decomposed into: “abnormality of [the] *other concept*” (e.g. HP:0000818 - abnormality of endocrine system). Not only can we add syntactic rules to reorder words, semantic synonyms of “abnormality”, such as “malformation” or “deformity”, can be added to express the concepts in similar ways. There are many other concepts that could benefit from recursively generating synonyms as the HPO appears to have compositional characteristics as well. There could also be subsets of rules depending on the context; recognizing concepts in doctor’s notes or electronic medical record will be expressed differently than those within the biomedical literature.

## Conclusions

In this work, we present a set of simple language generation rules to automatically generate synonyms for concepts in the Gene Ontology. These rules take into account the compositional nature of GO terms along with manually created syntactic and derivational variants derived from discussions with biologists, ontologists, and through analyzing Gene Ontology concepts as they are expressed within the literature. The 18 hand-crafted rules automatically generate over ∼1.5 million new synonyms for ∼66 % of all concepts within the Gene Ontology. We acknowledge the approach overgenerates synonyms, but we find that many generated synonyms do not appear within biomedical text, thus not hindering performance.

We argue that current synonyms in structured ontologies are insufficient for text-mining due to the vast degree of variability of expression within natural language text; our methods do not propose to solve this problem, but make a step in the right direction. This claim is supported through the examination of specific examples of concept variation in biomedical text and an empirical evaluation of the overlap of current GO synonyms and their expression in the CRAFT corpus.

We evaluate our synonym generation rules both intrinsically and extrinsically. Utilizing the CRAFT corpus for intrinsic evaluation, we evaluate three different sources of automatically generated synonyms 1) external ontology mappings, 2) recursive syntactic rules and 3) derivational variant rules. External mappings introduced too many false positives and are currently not recommended for use. The recursive syntactic rules added ∼194,000 new synonyms but did not significantly affect performance. Using a combination of recursive syntactic rules and derivational variant rules ∼300,000 new synonyms were generated, resulting in an increase in F-measure performance of 0.14, mostly due to greatly increased recall. This illustrates the importance of derivational variants for capturing natural expression.

Our rules were extrinsically evaluated on a large collection of one million full text documents. The rules aid in the recognition of ∼2,000 more unique concepts and increase the frequency in which all concepts are identified by 41 % over the baseline (Table 9), using only current information contained within the Gene Ontology. Specifically, the synonyms generated aid in the recognition of more complex and informative concepts. Manual validation of random samples conclude accuracy is not as high as desirable (74 %). An error analysis produced concrete next steps to increase the accuracy; simply removing one generation sub-rule, and filtering mentions with unmatched punctuation, increases accuracy of a random sample of 217 newly recognized concepts (∼1,450 mentions) to 83 %. Overall, manual analysis of 342 concepts (∼2,700 mentions) leads to an accuracy of 88 % (Additional file 4). We find that our rules increase the ability to recognize concepts from the Gene Ontology within the biomedical literature.

Even though we chose a specific dictionary based-system, ConceptMapper, to evaluate our rules, the generated synonyms can also be useful for many other applications. Any other dictionary based system can supplement its dictionary with the generated synonyms. Additionally, any machine learning or statistical based methods will be able to utilize the synonyms we generate to try to normalize the span of text identified as a specific entity type to an ontological identifier; this will provide a richer feature representation for target concepts. In addition, we provide examples of how these rules could generalize to other biomedical ontologies and discuss the impact of supercomputing on scaling this work.

Not only have our rules proven to be helpful for recognition of GO concepts, but there are also other applications separate from the evaluated task. They could be used to identify inconsistencies within the current Gene Ontology synonyms. Concepts that share similar patterns, i.e. *regulation of X*, should all contain synonyms that correspond to a certain syntactic pattern. While performing this work we identified a few concepts that should contain synonyms but do not, illustrating the usefulness of the presented rules for ontology quality assurance as originally outlined in Verspoor et al. [27]. Additionally, a certain conservative subset of our rules could easily be incorporated into TermGenie [24], a web application that automatically generates new ontology terms. Our rules would be of help to generate synonyms of the automatically generated concepts. It is our desire to submit the “good” synonyms identified within the text to the Gene Ontology Consortium for curation into the ontology. Additionally, there could possibly be a “text mining” synonym category added or we can deposit them, for the time being, within a larger application such as Freebase [43]. We would like other people to be able to use our synonyms for text mining so we provide the full list as Additional file 5.

## Additional files

Additional file 1 Full enumeration and explanation of each manually created rule. (PDF 363 kb)

Additional file 2 Outline of ConceptMapper parameters used for each branch of the Gene Ontology. (PDF 106 kb)

Additional file 3 Analysis of using external ontological mappings as synonyms. (PDF 165 kb)

Additional file 4 Detailed error analysis of manually reviewed concepts from large scale evaluation. (PDF 108 kb)

Additional file 5 The list of novel synonyms generated by this method. (TXT 129024 kb)
