# Supplementary material for: Gene Ontology synonym generation rules lead to increased performance in biomedical concept recognition
Source: J Biomed Semantics. 2016 Sep 9;7(1):52. doi: 10.1186/s13326-016-0096-7 (PMC5018193; doi:10.1186/s13326-016-0096-7)
Supplement: Additional file 1 — Full enumeration and explanation of each manually created rule. (PDF 363 kb) [file 13326_2016_96_MOESM1_ESM.pdf]

Enumeration of all rules and sub-rules for automatic generation of Gene Ontology synonyms

In this document we outline and enumerate the manually crafted synonym generation rules. There are three steps in automatically generating synonyms for Gene Ontology concepts. All three steps are repeated for each GO concept and each constituent term. The main idea behind the rules is to

1. Recursively decompose each Gene Ontology term to its constituent terms
2. Generate derivational variants for each of these constituent terms
3. Recombine all forms of all constituent terms (the constituent term itself, the generated derivational variants, and current synonyms of constituent term in the Gene Ontology) using differing syntactic and lexical rules

We first enumerate all rules and sub-rules used to generate synonyms, then walk through in detail a specific example of the rules applied to a more complex concept.

To recursively decompose each GO term, we utilize semi-frozen expressions as anchors to identify the constituent terms. Given the concept "GO:00006187 - dGTP biosynthetic process from dGDP", the constituent terms are "dGTP biosynthetic process" along with "dGDP", the first can be further broken down into a constituent term of "dGTP".

The words or phrases on the left hand side (*LHS*) of the concept are now referred to as *LHS* and words on the right hand side (*RHS*) are referred to as *RHS*; these can be replaced by both generated synonyms and the current synonyms in the ontology. Refering to the concepts above, "GO:00006187 - dGTP biosynthetic process from dGDP", would be decomposed using the *biosynthetic process term* rule. The *LHS* would correspond to "dGTP" and the *RHS* corresponds to "from dGDP". These are filled in using the rules described below. Here are two examples showing exactly how:

- "*LHS* synthesis *RHS*" ⇒ "dGTP synthesis from dGDP"
- "synthesis of *LHS RHS*" ⇒ "synthesis of dGTP from dGDP"

Recursive syntactic rules

The first step in all these rules is to decompose the to its most basic constituent terms by making sure the left and right hand side do not match any other rules. When no more rules are matched, syntactic synonyms are generated and then compositionally combined. [1]

[1] When "(-)" is seen within the rule, it signifies that this dash/space symbol is optional. For example, when we generate synonyms of "up(-)regulation", that entails "upregulation", "up regulation", and "up-regulation". Depending on how the text is tokenized and the dictionary handles punctuation, it is debatable if this is necessary, but nonetheless we generate them for completeness.

We now provide an enumeration of all semi-frozen anchors and what happens to the constituent terms found on the left hand side (*LHS*) and right hand side (*RHS*) of the anchor.

1. Dissection of terms containing "via" or "involved in"
    1.1. if preposition is "via"
        1.1.1. *LHS* via conversion to *RHS*
    1.2. if preposition is "involved in"
        1.2.1. *LHS* associated *RHS*
2. "regulation of" terms
    2.1. if preceded by "positive"
        2.1.1. positive regulation of *RHS*
        2.1.2. up(-)regulation of *RHS*
        2.1.3. activation of *RHS*
        2.1.4. *RHS* activation
        2.1.5. stimulation of *RHS*
        2.1.6. *RHS* stimulation
        2.1.7. promotion of *RHS*
        2.1.8. promote *RHS*
        2.1.9. *RHS* promotion
        2.1.10. induction of *RHS*
        2.1.11. *RHS* induction
        2.1.12. enhancement of *RHS*
        2.1.13. enhance *RHS*
        2.1.14. *RHS* enhancement
    2.2. if preceded by "negative"
        2.2.1. negative regulation of *RHS*
        2.2.2. down(-)regulation of *RHS*
        2.2.3. *RHS* down regulation
        2.2.4. anti(-)*RHS*
        2.2.5. repression of *RHS*
        2.2.6. *RHS* repression
        2.2.7. inhibition of *RHS*
        2.2.8. *RHS* inhibition
        2.2.9. suppression of *RHS*
        2.2.10. suppress *RHS*
        2.2.11. *RHS* suppression
3. "response to" terms
    3.1. if only *RHS*
        3.1.1. response to *RHS*
        3.1.2. *RHS* response
    3.2. if both *LHS* and *RHS*
        3.2.1. *LHS* response to *RHS*
        3.2.2. *RHS* responsible for *LHS*
        3.2.3. *RHS* resulting in *LHS*
    3.3. if *RHS* is an ion

- 3.3.1. *RHS*(-)responsive
- 3.3.2. *RHS*(-)response
- 3.3.3. *RHS* sensitivity
- 3.3.4. *RHS* resistance
- 3.3.5. *RHS* ion(-)responsive
- 3.3.6. *RHS* ion(-)response
- 3.3.7. *RHS* ion sensitivity
- 3.3.8. *RHS* ion resistance

4. “signaling” terms
    - 4.1. if *LHS* contains “receptor” *RHS* equals “pathway”
        - 4.1.1. *LHS* pathway
        - 4.1.2. *LHS* signaling
        - 4.1.3. *LHS* signalling
        - 4.1.4. *LHS* signaling pathway
        - 4.1.5. *LHS* signalling pathway
        - 4.1.6. *LHS* signaling process
        - 4.1.7. *LHS* signalling process
        - 4.1.8. *LHS* receptor signaling
        - 4.1.9. *LHS* receptor signalling
        - 4.1.10. *LHS* receptor signaling process
        - 4.1.11. *LHS* receptor signalling process
        - 4.1.12. *LHS* receptor pathway
        - 4.1.13. *LHS* receptor signaling pathway
        - 4.1.14. *LHS* receptor signalling pathway
    - 4.2. if *RHS* equals “patway”
        - 4.2.1. *LHS* pathway
        - 4.2.2. *LHS* signaling
        - 4.2.3. *LHS* signalling
        - 4.2.4. *LHS* signaling pathway
        - 4.2.5. *LHS* signalling pathway
        - 4.2.6. *LHS* signaling process
        - 4.2.7. *LHS* signalling process
        - 4.2.8. *LHS* receptor signaling
        - 4.2.9. *LHS* receptor signalling
        - 4.2.10. *LHS* receptor signaling process
        - 4.2.11. *LHS* receptor signalling process
        - 4.2.12. *LHS* receptor pathway
        - 4.2.13. *LHS* receptor signaling pathway
        - 4.2.14. *LHS* receptor signalling pathway

5. “biosynthetic process” terms
    - 5.1. if both *LHS* and *RHS*
        - 5.1.1. *LHS* biosynthesis *RHS*
        - 5.1.2. *LHS* biosynthesis pathway *RHS*
        - 5.1.3. biosynthesis of *LHS RHS*
        - 5.1.4. *LHS* synthesis *RHS*

5.1.5. synthesis of *LHS RHS*
5.1.6. *LHS* production *RHS*
5.1.7. *LHS* production pathway *RHS*
5.1.8. production of *LHS RHS*
5.1.9. *LHS* generation *RHS*
5.1.10. generation of *LHS RHS*
5.1.11. *LHS* formation *RHS*
5.1.12. formation of *LHS RHS*

5.2. if only *LHS*

5.2.1. *LHS* biosynthesis
5.2.2. *LHS* biosynthesis pathway
5.2.3. biosynthesis of *LHS*
5.2.4. *LHS* synthesis
5.2.5. synthesis of *LHS*
5.2.6. *LHS* production
5.2.7. *LHS* production pathway
5.2.8. production of *LHS*
5.2.9. *LHS* generation
5.2.10. generation of *LHS*
5.2.11. *LHS* formation
5.2.12. formation of *LHS*

5.3. if only *RHS*

5.3.1. biosynthesis *RHS*
5.3.2. biosynthesis pathway *RHS*
5.3.3. synthesis *RHS*
5.3.4. production *RHS*
5.3.5. generation *RHS*
5.3.6. formation*RHS*

6. "metabolic process" terms

6.1. if both *LHS* and *RHS*

6.1.1. *LHS* metabolism *RHS*
6.1.2. metabolism of *LHS RHS*

6.2. if only *LHS*

6.2.1. *LHS* metabolism
6.2.2. metabolism of *LHS*

6.3. if only *RHS*

6.3.1. metabolism *RHS*

6.4. if only "metabolic process"

6.4.1. metabolism

7. "catabolic process" terms

7.1. if both *LHS* and *RHS*

7.1.1. *LHS* catabolism *RHS*
7.1.2. catabolism of *LHS RHS*
7.1.3. *LHS* degradation *RHS*
7.1.4. degradation of *LHS RHS*

- 7.1.5. *LHS* breakdown *RHS*
- 7.1.6. breakdown of *LHH RHS*

7.2. if only *LHS*
- 7.2.1. *LHS* catabolism
- 7.2.2. catabolism of *LHS*
- 7.2.3. *LHS* degradation
- 7.2.4. degradation of *LHS*
- 7.2.5. *LHS* breakdown
- 7.2.6. breakdown of *LHH*

7.3. if only *RHS*
- 7.3.1. catabolism *RHS*
- 7.3.2. degradation *RHS*
- 7.3.3. breakdown *RHS*

7.4. if only "catabolic process"
- 7.4.1. catabolism

8. "binding"

8.1. if *LHS* and *RHS* equals "complex"
- 8.1.1. complex that bind *LHS*

8.2. if only *LHS*
- 8.2.1. binding of *LHS*
- 8.2.2. binds *LHS*
- 8.2.3. if *LHS* contains "receptor"
  - 8.2.3.1. *LHS*(-)binding receptor

9. "transport" terms

9.1. if *LHS* contains "transmembrane" and *RHS* equals "activity"
- 9.1.1. *LHS* transporter
- 9.1.2. transporter of *LHS*
- 9.1.3. transporting *LHS* transmembrane
- 9.1.4. transporting *LHS* across a membrane
- 9.1.5. transporting *LHS* across the membrane
- 9.1.6. transportation of *LHS* transmembrane
- 9.1.7. transportation of *LHS* across a membrane
- 9.1.8. transportation of *LHS* across the membrane
- 9.1.9. *LHS*

9.2. *LHS* and *RHS* equals "activity"
- 9.2.1. *LHS* transporter
- 9.2.2. transporter of *LHS*

10. "differentiation" terms

10.1. if only *LHS*
- 10.1.1. differentiation into *LHS*
- 10.1.2. differentiation into *LHS* cell

10.2. if *LHS* is found within Cell Ontology, grab all synonyms, *CLSYNS*
- 10.2.1. differentiation into *CLSYNS*
- 10.2.2. differentiation into *CLSYNS* cell
- 10.2.3. *CLSYNS* differentiation

11. “activity” terms
 11.1. if comma after “activity”
  11.1.1. *LHS* - *RHS*
  11.1.2. *LHS* that *RHS*
  11.1.3. *RHS LHS*
 11.2. if only *LHS*
  11.2.1. *LHS*

Derivational variant rules

For these rules we do not substitute phrases, but rather generate derivational variants for individual words. For input into these derivational rules we are assured these will be the most basic constituent terms. Because this is the case, we substitute *X* for capturing specific words and we can additionally specify which word in the concept we change *w1* or *w2* for the first and second word, respectively. Any of these can be substituted for the base form of the word, most likely a noun, or any of the derivational variants generated through WordNet [1] or lexical variant generator (LVG) [2], adjective or verb.

The PennTreebank part-of-speech (POS) tags are utilized in the enumeration of the derivational rules: NN = noun, VB = verb, JJ = adjective. All varying forms were converted to the basic POS tag, e.g. NNS = plural noun and were converted to NN. All these rules act on the constituent terms identified from the previous recursive syntactic rules.

1. Single word terms
 1.1. {NN} ⇒ {VB}
 1.2. {NN} ⇒ {JJ}
2. Double word terms
 2.1. {NN_1 NN_2} ⇒ {NN_1}, {VB_2 NN_1}, {JJ_1 NN_2}, {NN_1 JJ_2}
  2.1.1. *w2* of *w1*
  2.1.2. *w2* of a(n) *w1*
  2.1.3. if *w2* equals “development”
   2.1.3.1. specific development term ending in -genesis or -ization
  2.1.4. if *w2* is not a broad functional category(“binding”, “transport”, “secretion”,etc. . .)
   2.1.4.1. *w1*
  2.1.5. if *w2* equals “complex” and *w1* is not one of “mediator”, “receptor”, or “integrator”
   2.1.5.1. *w1*
  2.1.6. if *w2* equals “complex” and *w1* equals “immunoglobulin”
   2.1.6.1. antibody
   2.1.6.2. antibodies
   2.1.6.3. Ab
 2.2. {JJ_1 NN_2} ⇒ {JJ_1}, {JJ_1 JJ_2}

2.2.1. *w1 w2*

2.2.2. if *w2* equals "perception", "response", "region", "process" and *w1* does not equal "cellular"

2.2.2.1. *w1*

3. Triple word terms

3.1. {NN_1 NN_2 NN_3} ⇒ {NN_1 NN_3}, {NN_3 NN_1}, {VB_3}

3.1.1. *w3* of *w1 w2*

3.1.2. if *w2* equals "cell" or "nerve" and *w3* equals "morphogenesis" or "development"

3.1.2.1. *w1 w3*

3.1.2.2. *w3* of *w1*

3.1.3. if *w1* equals "cell" and *w3* does not equal specific terms associated with cells such as "site", "determination", "formation", "assembly", etc...

3.1.3.1. *w3*

3.1.3.2. *w1 w3*

3.1.3.3. *w3* of *w1*

4. "cell part" terms

4.1. if concept has parent of "cell part" or "organelle part", *RHS* corresponds to specific part of cell

4.1.1. *LHS RHS*

4.1.2. *RHS* of *LHS*

5. "sensory perception" terms

5.1. generate other forms of *w4*, i.e. "taste"⇒"gustory"

6. "transcription, *X*-dependent" terms

6.1. *X*(-)reverse transcription

6.2. *X*(-)RT

6.3. *X*(-)dependent reverse(-)transcription

6.4. *X*(-)dependent RT

6.5. if *X* equals RNA

6.5.1. reverse(-)transcription

6.5.2. RT

7. "*X* strand annealing activity" terms

7.1. *X* annealing

7.2. *X* hybridization

7.3. annealing

7.4. hybridization

In Figure A1 we provide an example of utilizing our rules for a more complex and difficult to recognize term, "GO:0061005 - cell differentiation involved in kidney development". The original concept is first decomposed through the recursive *terms containing preposition* rule; both sides of the prepositional phrase, "involved in", will be decomposed further if possible. The left hand side, "GO:0030154 - cell differentiation", can be decomposed using the syntactic *differentiation terms* rule and other synonyms will be generated using the

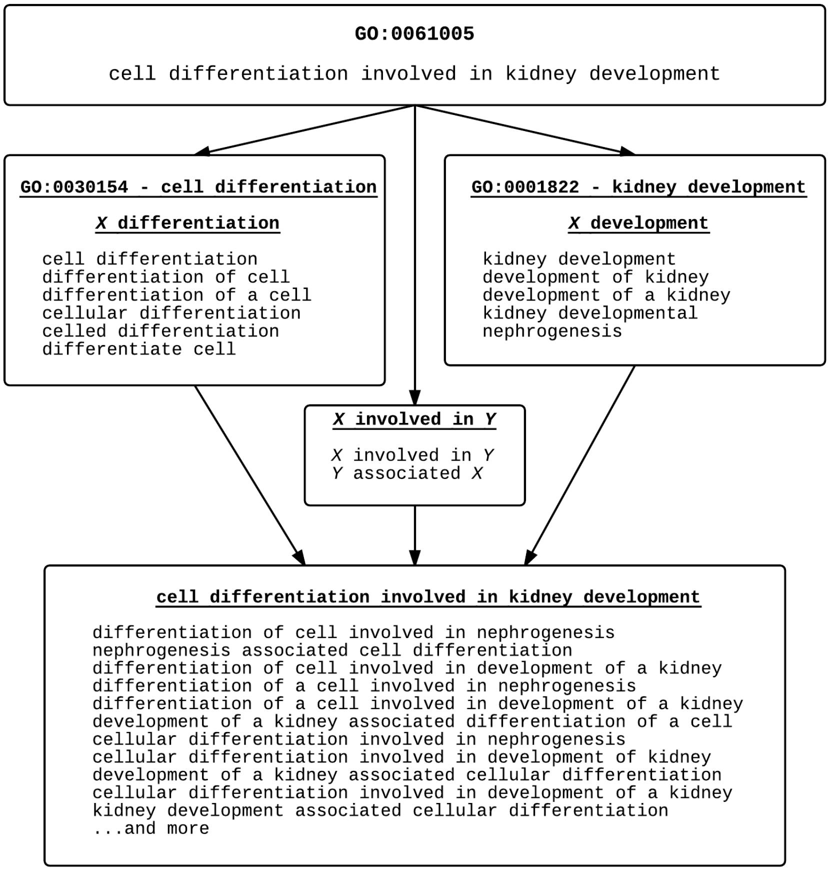

Figure A1: **Syntactic and derivational synonyms generation example.** A single GO concept broken down into its composite parts (bolded and underlined), synonyms generated for each part (text underneath the part), then combination of all synonyms from all composite parts to form complete synonym of the original concept.

double word derivational rule; the first three synonyms listed in the example are from "differentiation" while the last three are generated from derivations of the words "cell" and "differentiation". The right hand side, "GO:0001822 - kidney development", synonyms are generated solely from the *double word* rule. All synonyms generated for the left and right side are compositionally combined with the varying ways to express "involved in" to generate 60 synonyms for the original concept.

References

[1] Fellbaum, C.: WordNet: An Electronic Lexical Database (Language, Speech, and Communication). The MIT Press, Cambridge, Massachusetts (1998). http://www.amazon.ca/exec/obidos/redirect?tag=citeulike04-20&path=ASIN/026206197X

[2] NLM: LVG:Lexical variant generatior. http://lexsrv2.nlm.nih.gov/LexSysGroup/Projects/lvg/2012/web/index.html (2012)
