# Supplementary material for: Gene Ontology synonym generation rules lead to increased performance in biomedical concept recognition
Source: J Biomed Semantics. 2016 Sep 9;7(1):52. doi: 10.1186/s13326-016-0096-7 (PMC5018193; doi:10.1186/s13326-016-0096-7)
Supplement: Additional file 2 — Outline of ConceptMapper parameters used for each branch of the Gene Ontology. (PDF 106 kb) [file 13326_2016_96_MOESM2_ESM.pdf]

Parameters used for each sub-branch of the Gene Ontology

Table A1: **Summarization of ConceptMapper parameters.**

Parameter	Description	MF	BP	CC
Search strategy	CONTIGUOUS - returns longest match of contiguous tokens in the span, SKIP_ANY - returns longest match of not-necessarily contiguous tokens in the span, SKIP_ANY_ALLOW_OVERLAP - returns longest match of not-necessarily contiguous tokens in the span, this implies orderIndLookup	CONTIGUOUS	CONTIGUOUS	CONTIGUOUS
Case match	IGNORE - fold everything to lowercase more matching, INSENSITIVE - fold only tokens with initial caps to lowercase, SENSITIVE - performs no case folding, FOLD_DIGIT -fold only (and only) tokens with a digit	IGNORE	INSENSITIVE	INSENSITIVE
Stemmer	specifics which stemmer to use - PORTER, BIOLEMMATIZER, or NONE	BIOLEMMATIZER	PORTER	PORTER
Stop words	a list of stopwords to remove - PUBMED or NONE	NONE	NONE	NONE
Order independent lookup	if set to TRUE token ordering within the sentence is ignored ("box top" would match"top box") - TRUE or FALSE	FALSE	FALSE	FALSE
Find all matches	If TRUE all dictionary matches within the sentence are returned, otherwise only the longest is returned - TRUE or FALSE	FALSE	TRUE	FALSE
Synonyms	specifies which synonyms will be included when making the dictionary - EXACT_ONLY or ALL	EXACT_ONLY	ALL	EXACT_ONLY
