# Supplementary material for: Gene Ontology synonym generation rules lead to increased performance in biomedical concept recognition
Source: J Biomed Semantics. 2016 Sep 9;7(1):52. doi: 10.1186/s13326-016-0096-7 (PMC5018193; doi:10.1186/s13326-016-0096-7)
Supplement: Additional file 3 — Analysis of using external ontological mappings as synonyms. (PDF 165 kb) [file 13326_2016_96_MOESM3_ESM.pdf]

Mappings between ontologies and other biomedical resources

The first source of synonyms we used were those linked directly to Gene Ontology concepts through manually curated external mappings. We imported synonyms from four different sources, the Brenda database (Enzyme Commission numbers), UniProt knowledgebase, UniProt subcellular localization, and Wikipedia. Each of these resources contains classes or entities that are manually assigned indexes to identical, similar, or related GO terms (`http://geneontology.org/page/download-mappings`). It is noted by the GO Consortium that the mappings should not be taken as exact or complete. For both UniProt and Wikipedia, the official mappings were downloaded from the GO mapping website while Brenda was accessed through the provided SOAP server; all synonyms linked to the GO concepts were added as synonyms and re-evaluated on the CRAFT corpus.

Table A1: **Results for each external mapping source on the CRAFT corpus.**

Method	Synonyms added	Affected terms	TP	FP	FN	P	R	F
**Cellular Component**								
Baseline (B1)	X	X	5,532	452	2,822	0.925	0.662	**0.772**
Baseline (B2)	X	X	5,532	452	2,822	0.925	0.662	**0.772**
Brenda (EC)	0	0	5,532	452	2,822	0.925	0.662	**0.772**
UniProt	348	330	5,547	709	2,807	0.887	0.664	0.759
Wikipedia	210	210	5,519	1,014	2,835	0.845	0.661	0.742
All Combined	471	419	5,534	1,271	2,820	0.813	0.662	0.730
**Molecular Function**								
Baseline (B1)	X	X	337	146	3,843	0.698	0.081	0.145
Baseline (B2)	X	X	1,772	964	2,408	0.648	0.424	**0.512**
Brenda (EC)	22,158	2,870	1,768	2,773	2,412	0.389	0.423	0.406
UniProt	111	105	1,773	2,608	2,407	0.404	0.424	0.414
Wikipedia	31	31	1,772	2,666	2,408	0.399	0.424	0.411
All Combined	22,258	3,006	1,773	3,015	2,411	0.370	0.424	0.395
**Biological Process**								
Baseline (B1)	X	X	4,909	5,682	12,004	0.464	0.290	0.357
Baseline (B2)	X	X	4,913	5,951	12,000	0.452	0.291	0.354
Brenda (EC)	0	0	4,913	5,951	12,000	0.452	0.291	0.354
UniProt	361	346	5,392	7,120	11,521	0.431	0.319	**0.367**
Wikipedia	343	338	4,969	6,227	11,944	0.444	0.294	0.354
All Combined	660	600	5,440	7,396	11,473	0.424	0.322	0.366
**All Gene Ontology**								
Baseline (B1)	X	X	10,778	6,280	18,669	0.632	0.366	0.464
Baseline (B2)	X	X	12,217	7,367	17,230	0.624	0.415	**0.498**
Brenda (EC)	22,158	2,870	12,213	9,176	17,234	0.571	0.415	0.480
UniProt	720	781	12,712	10,437	16,735	0.549	0.432	0.483
Wikipedia	584	579	12,260	9,907	17,187	0.553	0.416	0.475
All Combined	23,389	4,025	12,747	11,682	16,704	0.522	0.433	0.473

The results of each external synonym source, broken down by sub-branch of GO, can be seen in Table A1. Overall, for the CC and MF branches, we find

that no external mappings increased performance over the baseline because of a large decrease in precision (P) without a corresponding increase in recall (R). With respect to the BP branch, we find a slight, 0.01, improvement from baselines in overall performance when using synonyms from UniProt. This slight improvement comes from a 0.03 increase (483 more true positives) in R and a 0.03 decrease (1,438 more false positives) in P. An error analysis was performed on the many false positives introduced through using external mappings but unfortunately no systematic method to improve or filter them was discovered (data not shown). Overall, based upon these results, external mappings introduce significantly more errors than correctly recognized concepts and are not suggested to be useful as a whole for concept recognition.

With more analysis, it is possible that filters could be created to reduce the false positives before external synonyms are inserted into the dictionary, but we leave that towards future work. Additionally, we are aware that there are many other sources of external mappings that each need to be examined individually to evaluate their usefulness as synonyms.
