# Supplementary material for: Gene Ontology synonym generation rules lead to increased performance in biomedical concept recognition
Source: J Biomed Semantics. 2016 Sep 9;7(1):52. doi: 10.1186/s13326-016-0096-7 (PMC5018193; doi:10.1186/s13326-016-0096-7)
Supplement: Additional file 4 — Detailed error analysis of manually reviewed concepts from large scale evaluation. (PDF 108 kb) [file 13326_2016_96_MOESM4_ESM.pdf]

Error analysis of Gene Ontology mentions

There were three main types of errors introduced by our synonym generation rules.

1. Naive stemming introducing incorrect concepts
2. Incorrect level of specificity due to information loss
3. Inclusion of incorrect punctuation

Some of these are also seen in the baseline evaluation. We explain and provide examples of each type then re-evaluate after a simple fix.

The most common type of error makes up 60% (225 out of 377) of all errors and is introduced through stemming during the concept recognition step. One of our syntactic recursive rules, *regulation of* terms, has a syntactic variant of *X activation*, which keeps the same semantics as the original concept. The error is introduced by using a stemmer within our concept recognition pipeline, that has been shown to increase performance [1], but because both the words "activation" and "activity" stem to "activ" there are many incorrect spans of *X activity* normalized to the *positive regulation* concept identifier. For example, our rules add a synonym of "collagen binding activation" to the concept "GO:0033343 - positive regulation of collagen binding". Because of the stemmer, many spans of "collagen binding activity" are grounded to GO:0033343, which is incorrect. "activation"⇒"activity" makes up a majority of the errors, but we also find the text span "importance of glycine" grounded to "GO:0036233 - glycine import" due to the rule generated synonym "import of glycine". These errors could be removed by not including the stemmer in the dictionary lookup or employing a stemmer that handles these words in a more linguistically sensitive manner. It is unclear what other effects that would have on the other concepts identified. We plan on exploring the impact of different parameter combinations with these new synonym generation rules.

The second most common type of error, making up 25% (95 out of 377), is due to synonyms being generated at differing levels of specificity; this can occur during information loss or recursively using narrow/broad defined synonyms within the ontology. Currently our rules treat all synonyms for a concept the same (this can be changed quite easily) and when these other types of related synonyms are incorporated through the recursive syntactic rules they can introduce varying levels of specificity. For example, the text spans in the large corpus "anti-ryanodine receptor" and "inhibition of ryanodine receptors" are identified to be of concept "GO:0060315 - negative regulation of ryanodine-sensitive calcium-release channel activity". We believe that these errors are due to incorporating current synonyms of different specificities, i.e. broad or narrow synonyms within the Gene Ontology. All of these mentions are *related* to the concept identifier they are normalized to, but not an *exact* synonym. Many of these errors can be judged as partially correct; the use of a hierarchical precision and hierarchical recall metric in a comparison to a gold standard would give such partial credit [2, 3, 4].

The least common type of error seen at only 15% (57 out of 377) is due to incorrect punctuation being incorporated into the text span. These are most likely due to our tokenizer performing poorly in the parsing of particular situations. Some errors appear to come from information derived from tables. These could be removed through a post-processing filter – e.g. any mentions with semicolons or colons, unmatched parenthesis or quotes could be removed as these are often tokenized incorrectly. The more difficult types of punctuation to filter are those containing commas. For example, the concept "GO:2001170 - negative regulation of ATP biosynthetic process" is recognized within the sentence "These include cessation of **ATP synthesis, inhibition** of respiration, and a drop in $\Delta\Psi$." (PMCID: PMC2683183); it is evident to a human that this is incorrect, but without the comma the span "ATP synthesis inhibition" appears to be correct. Since punctuation other than sentence boundaries is ignored during matching, such sentences can result in false positive matches.

To help reduce these errors we implemented two simple strategies: 1) Removed all text-spans that mention containing unmatched parenthesis, semicolons, or colons and 2) removed the specific rule within the *regulation of* rule that generates the *X activation* synonyms. We refer to this new set of mentions produced as the *corrected* set. In total there were ∼850,000 likely erroneous mentions removed through these two observations. After manual re-validation we found that accuracy increased from 0.74 to 0.82 for these 217 concepts and increased from 0.83 to 0.88 when aggregated over all 342 concepts manually examined. Through error analysis, we have shown that the accuracy of our rules can be improved using only very simple techniques, however, we believe we can achieve much higher accuracy through future work by incorporating syntactic parses along with more detailed analysis and refinement of the current rules.

References

[1] Funk, C., Baumgartner, W., Garcia, B., Roeder, C., Bada, M., Cohen, K., Hunter, L., Verspoor, K.: Large-scale biomedical concept recognition: an evaluation of current automatic annotators and their parameters. BMC Bioinformatics **15**(1), 59 (2014)

[2] Verspoor, K., Cohn, J., Mniszewski, S., Joslyn, C.: A categorization approach to automated ontological function annotation. Protein Science **15**(6), 1544–1549 (2006)

[3] Clark, W.T., Radivojac, P.: Information-theoretic evaluation of predicted ontological annotations. Bioinformatics **29**(13), 53–61 (2013)

[4] Bada, M., Baumgartner Jr, W., Funk, C., Hunter, L., Verspoor, K.: Semantic precision and recall for concept annotation of text. In: Proceedings of the BioOntologies SIG at ISMB'14 (2014)
